# Posterior spinal instrumentation and decompression with or without cross-link?

**DOI:** 10.1016/j.xnsj.2021.100093

**Published:** 2021-11-17

**Authors:** Marco D. Burkhard, Frédéric Cornaz, José Miguel Spirig, Florian Wanivenhaus, Rafael Loucas, Marie-Rosa Fasser, Jonas Widmer, Mazda Farshad

**Affiliations:** a Department of Orthopedics, Balgrist University Hospital, University of Zurich, Switzerland; b University Spine Center Zürich, Balgrist University Hospital, University of Zurich, Switzerland; c Institute for Biomechanics, Balgrist Campus, ETH Zurich, Zurich, Switzerland; d Spine Biomechanics, Department of Orthopedic Surgery, Balgrist University Hospital, University of Zurich, Switzerland

**Keywords:** Cross-link, Cross-connector, Spine, lumbar, Instrumentation, Segmental stability, Biomechanical

## Abstract

**Background:**

Posterior lumbar instrumentation requires sufficient primary stiffness to ensure bony fusion and to avoid pseudarthrosis, screw loosening, or implant failure. To enhance primary construct stiffness, transverse cross-link (CL) connectors attached to the vertical rods can be used. Their effect on the stability of a spinal instrumentation with simultaneous decompression is yet not clear. This study aimed to evaluate the impact of CL augmentation on single-level lumbar instrumentation stiffness after gradual decompression procedures.

**Methods:**

Seventeen vertebral segments (6 L1/2, 6 L3/4, 5 L5/S1) of 12 fresh-frozen human cadavers were instrumented with a transpedicular screw–rod construct following the traditional pedicle screw trajectory. Range of motion (ROM) of the segments was sequentially recorded before and after four procedures: (A) instrumented before decompression, (B) instrumented after unilateral laminotomy, (C) instrumented after midline bilateral laminotomy, and (D) instrumented after unilateral facetectomy (with transforaminal lumbar interbody fusion [TLIF]). Each test was performed with and without CL augmentation. The motion between the cranial and caudal vertebrae was evaluated in all six major loading directions: flexion/extension (FE), lateral bending (LB), lateral shear (LS), anterior shear (AS), axial rotation (AR), and axial compression/distraction (AC).

**Results:**

ROM was significantly reduced with CL augmentation in AR by Δ0.03–0.18° (7–12%) with a significantly higher ROM reduction after more extensive decompression. Furthermore, slight reductions in FE and LB were observed; these reached statistical significance for FE after facetectomy and TLIF insertion only (Δ0.15; 3%). The instrumentation levels did not reveal any subgroup differences.

**Conclusion:**

CL augmentation reduces AR-ROM by 7–12% in single-level instrumentation of the lumbar spine, with the effect increasing along with the extensiveness of the decompression technique. In light of the discrete absolute changes, CL augmentation may be warranted for highly unstable vertebral segments rather than for standard single-level posterior spinal fusion and decompression.

## Background

Spinal instrumentation with transpedicular screw–rod constructs is the gold standard in posterior fusion surgery, with an increasing number of procedures performed every year [1,2]. The success of this technique is related to increased primary stability of the construct [3]. To further enhance the construct stiffness, transverse cross-link (CL) connectors bilaterally attached to the vertical rods or screw heads can augment the stiffness of the transpedicular system of spinal instrumentation.

While some studies have demonstrated an increased primary stiffness [4,5], others have denied any significant gain in stiffness due to CL augmentation [6,7]. The potential application of CL is particularly interesting for situations in which the spine is considerably destabilized, for example after a corpectomy or pedicle subtraction osteotomy [8,9].

However, the effects of CL augmentation have not been investigated after gradual decompression surgery, up to now. Therefore, the literature does not contain any guidelines or best practices for the use of CL augmentation. The decision still relies on the treating surgeon's subjective intraoperative estimation.

The purpose of the present study was to investigate the impact of CL augmentation on primary stiffness in a single-level pedicle screw instrumentation construct in the lumbar spine after gradual decompression procedures.

## Methods

### Specimen preparation and instrumentation

This study was approved by the local ethical committee (BASEC Nr. 2017-00874). Seventeen vertebral segments (6 L1/2, 6 L3/4, 5 L5/S1) of 12 fresh-frozen human spine cadavers (Science Care, Phoenix, AZ, USA) with an average age of 59 years (range 50–68 years; 8 males and 3 females) were used for this study. Apart from age-appropriate changes, the specimens were free of any osseous defects or deformities, as computed tomography (CT) and magnetic resonance imaging (MRI) scans illustrated. Intervertebral discs, facet joints, and interspinous ligaments were intact in all specimens. Following the initial dissection, the specimens were stored at −20 °C until instrumentation, mounting, and biomechanical testing; these procedures were performed after the specimens were thawed overnight at average room temperature (approximately 20 °C).

All pedicle screw entry points and trajectories were planned with computer-aided design (CAD) software. 2.7 mm drill guides were 3D printed and used for instrumentation with cannulated titanium alloy polyaxial pedicle screws (Medacta International, Castel San Pietro, Switzerland). Optimized screw length and diameter were planned with the CAD software for each vertebra and pedicle based on the 3D-planning and ranged between 40 - 55 mm and 5.0 - 7.0 mm, respectively. Prebent titanium rods were used to vertically link the pedicle screws on each side. One CL (straight cross-connector, MUST Medacta International, Switzerland) was mounted horizontally in the center of the two rods and locked with a 5.5 Nm torque according to the standard surgical technique. After instrumentation, the segments were cranially and caudally fixed with 3D-printed clamps, allowing for free movement within the segments for biomechanical testing [10].

### Biomechanical setup

**Fig. 1:**
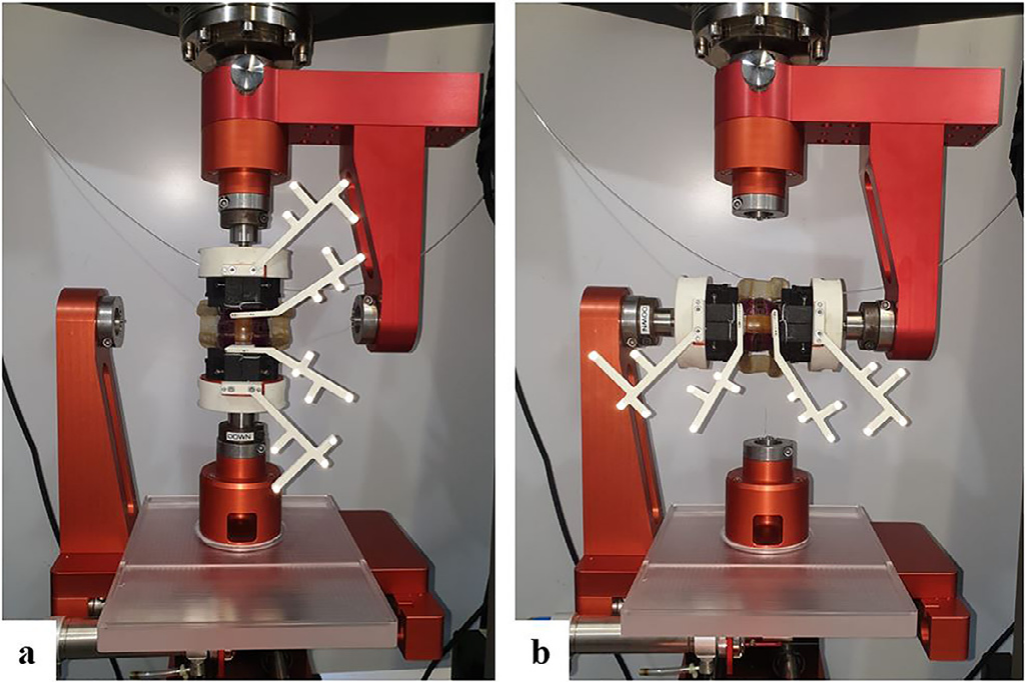
**Biomechanical setup.** A) Functional spine unit–Here, a sawbones model–In the vertical position for testing of axial rotation and axial compression. B) Spine segment installed in the horizontal position to measure flexion/extension and lateral shear. The caudal vertebra was attached to the semiconstrained test rig, which allowed for free translational movements in the horizontal plane. The cranial and caudal vertebral bodies were each installed with a marker, and additional markers were set on the cranial and caudal 3D-printed mounting clamps to control for excess movement between the vertebrae and the mounting system.

The stiffness of the specimens was tested in six major axes: flexion/extension (FE), lateral bending (LB), anterior shear (AS), lateral shear (LS), axial compression/distraction (AC), and axial rotation (AR). A biaxial linear-torsion testing protocol (Zwick/Roell Allroundline 10kN and testXpert III Software, ZwickRoell GmbH & Co. KG, Germany) was used; a predefined load was applied to the cranial vertebra, while the caudal vertebra was fixed to the semiconstrained test rig. The setup was designed to allow free translational movements perpendicular to the loading direction (Fig. 1) [11,12]. FE, LB, and AR were performed with an angular velocity of 1°/sec until a torque of ±7.5 Nm was reached. AS and LS were performed with 0.5 mm/sec to ±150 N and AC with 0.1 mm/sec to +400 N compression and −150 N distraction [13]. After five preconditioning cycles, the range of motion (ROM) of the vertebral segment was recorded over one cycle with a motion-capturing system with 10 Hz and 0.09 mm accuracy (Fusion Track 500, Atracsys, Puidoux, Switzerland). Data were recorded over the whole loading cycle, and the amplitude of the translational movement of the markers (LS, AS, AR) and the projected angulation in the motion plane (FE, LB, and AR) were evaluated.

### Biomechanical evaluation

**Fig. 2:**
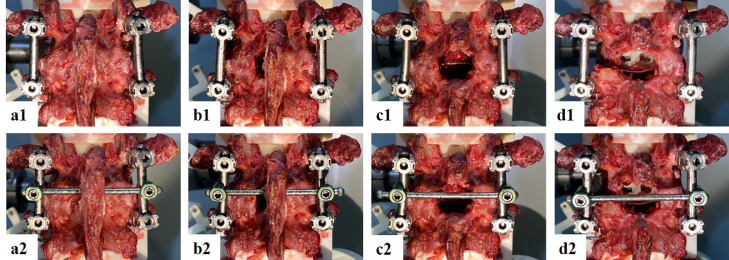
**Cross-link augmentation after varying degrees of surgical decompression.** A = intact, B = unilateral laminotomy, C = midline decompression, D = unilateral facetectomy and insertion of a transforaminal interbody fusion cage. 1 = instrumentation without cross-link, 2 = instrumentation with cross-link.

Segmental ROM was tested with and without a CL before and after three sequential types of decompression surgeries: (A) native = no decompression, (B) microsurgical decompression with unilateral laminotomy, (C) midline decompression with bilateral laminotomy, and (D) unilateral facetectomy and insertion of a transforaminal lumbar interbody fusion cage (TLIF; MectaLIF Transforaminal, Medacta International, Switzerland; Fig. 2). The correct TLIF cage size was determined based on the previously acquired CT scans. Prior to cage implantation, segmental distraction of −100 N was applied to facilitate cage insertion. An axial compression of +200 N was applied before tightening the vertical rods after cage insertion. Instrumentation and decompression procedures were performed by an experienced spine surgeon.

### Statistical evaluation

The statistical evaluation was performed with MATLAB (Matlab 2019a, MathWorks, Massachusetts, USA). A Shapiro–Wilk test showed some of the data to be nonnormally distributed. Therefore, nonparametric statistical testing was performed. Statistical evaluations of the differences in medians between various instrumentation and decompression techniques were carried out. The Mann–Whitney U test and the Wilcoxon signed-rank test were used to compare unmatched and matched data, respectively. Due to multiple comparisons, the significance level α was adjusted with Bonferroni corrections (α = 0.0125).

## Results

**Fig. 3:**
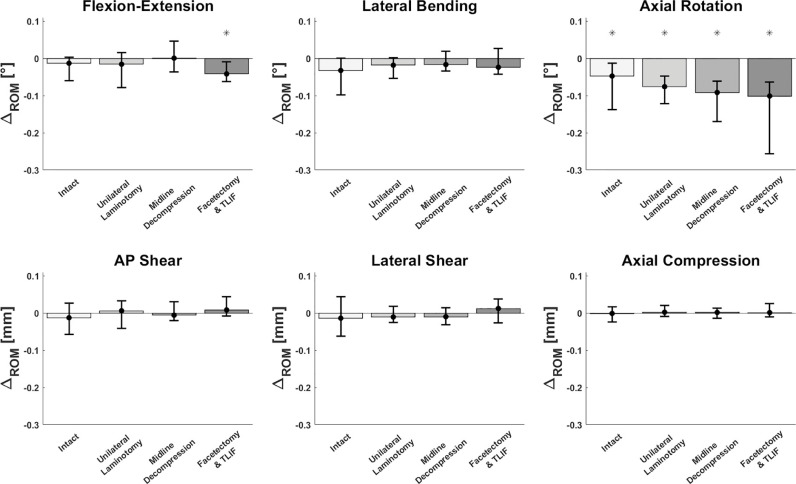
**Absolute reduction in range of motion (ROM) after cross-link augmentation.** Change is calculated as Δ_ROM_ = ROM_Crosslink_ − ROM_Instrumentation_. The medians of the single differences are shown, along with the 25^th^ and 75^th^ percentiles. Bars below 0 indicate less motion with than without a cross-link. Asterisks (*) indicate statistical significance.

The addition of a CL to the posterolateral pedicle screw fusion construct significantly reduced the ROM in AR (Fig. 3) before and after all decompression techniques. The absolute and relative reduction in AR-ROM due to CL augmentation was 0.03–0.18° and 7–12%, respectively (Table 1). The more extensive the decompression technique was, the greater was the observed absolute AR reduction, with a significant difference after unilateral facetectomy and TLIF insertion compared to after unilateral laminotomy (*p* = 0.0086).

**Table 1:** Absolute range of motion (ROM) and relative reduction of ROM due to cross-link augmentation.

	Instrumentation (I)		Cross-link (CL)		1-CL/I (%)		p-Value
	Median	25^th^ – 75^th^	Median	25^th^ – 75^th^	Median	25^th^ – 75^th^	
**Intact**							
FE (°)	0,95	0,66 - 1,65	0,95	0,71 - 1,60	1,25	(-0,25) - 4,98	0,093
LB (°)	1,53	1,29 - 1,79	1,53	1,30 - 1,79	2,26	(-0,05) - 5,20	0,015
AR (°)	**0.89**	**0,70 - 1,22**	**0,86**	**0,69 - 1,06**	**7,11**	**1,69 - 12,78**	**<0,001**
AS (mm)	1,03	0,81 - 1,19	0,94	0,75 - 1,18	1,68	(-2,19) - 5,77	0,332
LS (mm)	1,00	0,66 - 1,24	0,95	0,61 - 1,26	1,44	(-4,04) - 6,30	0,309
AC (mm)	0,28	0,21 - 0,49	0,27	0,17 - 0,49	1,20	(-4,08) - 7,18	0,438
**Unilateral Laminotomy**							
FE (°)	1,02	0,73 - 1,62	1,06	0,76 - 1,60	1,63	(-2,25) - 4,26	0,136
LB (°)	1,53	1,32 - 1,88	1,41	1,26 - 1,83	1,24	(-0,14) - 2,87	0,031
AR (°)	**1,04**	**0,79 - 1,17**	**0,90**	**0,76 - 1,04**	**7,61**	**6,18 - 10,03**	**<0,001**
AS (mm)	0,97	0,83 - 1,43	0,95	0,79 - 1,40	(-0,90)	(-3,57) - 3,45	0,906
LS (mm)	1,07	0,73 - 1,27	1,04	0,70 - 1,26	0,85	(-1,40) - 2,95	0,619
AC (mm)	0,29	0,22 - 0,49	0,28	0,23 - 0,49	(-0,83)	(-5,09) - 6,72	0,756
**Midline Decompression**							
FE (°)	1,09	0,77 - 1,79	1,13	0,81 - 1,79	(-0,09)	(-3,13) - 2,29	0.831
LB (°)	1,67	1,38 - 1,88	1,67	1,39 - 1,86	0,97	(-1,40) - 2,15	0,381
AR (°)	**1,06**	**0,82 - 1,26**	**0,91**	**0,76 - 1,12**	**11,76**	**8,03 - 12,87**	**<0,001**
AS (mm)	1,05	0,84 - 1,47	1,04	0,78 - 1,47	0,54	(-1,83) - 2,19	0,868
LS (mm)	1,09	0,76 - 1,37	1,10	0,77 - 1,40	0,68	(-1,86) - 3,68	0,435
AC (mm)	0,29	0,23 - 0,50	0,31	0,23 - 0,50	(-0,05)	(-4,29) - 4,37	1,00
**Facetectomy & TLIF**							
FE (°)	**1,46**	**1,01 - 1,90**	**1,31**	**1,01 - 1,77**	**2,81**	**1,10 - 4,89**	**0.004**
LB (°)	1,59	1,02 - 2,06	1,56	1,01 - 2,04	1,45	(-1,28) - 4,24	0,266
AR (°)	**1,23**	**1,01 - 1,56**	**1,05**	**0,92 - 1,36**	**10,21**	**7,32 - 15,76**	**<0,001**
AS (mm)	0,95	0,77 - 1,66	0,94	0,83 - 1,67	(-0,46)	(-4,58) - 1,04	0,210
LS (mm)	0,93	0,71 - 1,66	0,87	0,73 - 1,63	(-0,92)	(-3,81) - 2,25	0,723
AC (mm)	0,31	0,17 - 0,60	0,30	0,18 - 0,62	(-0,51)	(-6,02) - 2,53	0,469

ROM = range of motion. 25th-75th = 25% and 75%. FE = flexion/extension, LB = lateral bending, AR = axial rotation, AS = anterior shear, LS = lateral shear, AC = axial compression. TLIF = transforaminal interbody fusion. Bold type indicates statistical significance

A significant reduction in ROM following CL augmentation was further observed in FE after facetectomy and TLIF insertion, with absolute and relative changes of 0.15° and 3%, respectively (*p* = 0.004). FE was not significantly affected following the other decompression techniques.

A trend toward a slight reduction in LB following CL augmentation was also observed after use of all decompression techniques. However, these changes did not reach statistical significance. No ROM reduction was observed in the translational motions AS, LS, and AC after use of all decompression techniques.

Subgroup analyses of the instrumentational levels L1/2, L3/4, and L5/S1 confirmed a reduction in AR-ROM or all instrumentation levels, with a trend toward higher reductions after more extensive decompression, but these differences did not reach statistical significance in the subgroups with five to six specimens apiece (Table 2).

**Table 2:** **Subgroup analysis of different fusion segments.** The relative impact of adding a cross-link on range of motion is calculated as Δ_ROM_ = 1 - ROM_Crosslink_ / ROM_Instrumentation_ [%].

	L1/2 (n=6)		L3/4 (n=6)		L5/S1 (n=5)	
	Δ_ROM_ [%]	p-Value	Δ_ROM_ [%]	p-Value	Δ_ROM_ [%]	p-Value
Flexion-Extension						
Intact	0.67	0.563	-0.17	1.000	3.07	0.063
Unilateral Laminotomy	1.66	0.563	0.34	0.844	1.63	0.313
Midline Decompression	-1.37	0.312	1.07	0.563	0.76	1.000
Facetectomy & TLIF	2.30	0.156	3.09	0.219	2.68	0.125
Lateral Bending						
Intact	2.45	0.063	1.06	0.438	2.54	0.313
Unilateral Laminotomy	0.60	0.156	0.66	0.438	2.66	0.313
Midline Decompression	0.90	0.688	0.69	0.844	1.18	0.625
Facetectomy & TLIF	0.43	0.844	0.30	0.844	3.78	0.063
Axial Rotation						
Intact	3.20	0.094	9.60	0.031	12.74	0.063
Unilateral Laminotomy	8.29	0.031	6.25	0.031	9.06	0.063
Midline Decompression	12.99	0.031	9.05	0.063	11.05	0.063
Facetectomy & TLIF	14.55	0.031	6.35	0.156	14.90	0.063

ROM = range of motion. TLIF = transforaminal interbody fusion. Boldfacetype indicates statistical significance

## Discussion

This biomechanical study provides comprehensive insight into the impact of CL augmentation on a single-level lumbar segment after posterior pedicle screw instrumentation and use of various decompression techniques. The main finding is that CL augmentation of a lumbar single-level fusion construct leads to a decrease in AR-ROM of 7–12%, increasing with the scope of the decompression surgery.

While several studies have been published on the biomechanical impact of CL augmentation, differences in testing methods—and in instrumentation and destabilization techniques—complicate direct comparisons between studies [14]. Additionally, the relatively small sample sizes and the nonhuman test models often employed in previous studies relativize the power and translatability into clinical practice [5,8,9,15,16]. Therefore, it is not surprising that the literature has reported a broad array of results. Our study potentially allows for more profound conclusions on this topic due to the comparatively large sample (*n* = 17), the human lumbar cadaveric model, and the standardized instrumentation and decompression techniques.

Our study reveals that CL augmentation was significantly more effective after unilateral facetectomy and TLIF insertion compared to CL use in a nondecompressed segment or CL use in unilateral or bilateral laminotomy alone. This outcome highlights the potential of CL augmentation in rotatory unstable contexts. Chutkan et al. [17] have reported similar results: a decrease in primary stiffness after facetectomy. However, due to only a minimal gain in stiffness, the authors concluded that, based on their data, CL augmentation is not justified in single-level instrumentation. Lim et al. [18], implementing horizontal CL augmentation in calf cadaver specimens, reported notably increased stiffness in AR and LB of 13.9% and 15.7%, respectively. In contrast, in the present study, the effect of CL augmentation on LB was only minimal and nonsignificant.

In light of the absolute ROM decrease with CL augmentation of only 0.1–0.2° in AR, the question arises of whether this gain in primary stiffness is of great clinical relevance. Previous biomechanical investigations have shown that the pedicle screw–rod construct alone leads to an increase in primary stability of 44–52% in AR compared to that seen in uninstrumented segments [19,20]. The present study's relative reduction of AR-ROM of 7–12% following CL augmentation would thus only lead to an additional AR-ROM reduction of 3–6% relative to the uninstrumented segment (7–12% of 44–52%). Therefore, whether CL augmentation and the subsequent AR reduction pivotally improve the biological and biomechanical environment for bone growth remains questionable.

Some remaining micromotion in the instrumented segment could even be beneficial; per Wolff's law, bone formation is stimulated by mechanical loading [21]. Bone growth following spinal fusion surgery is intuitively similar to fracture healing, in which micromotion through flexible fixation and dynamization of the implants can lead to faster and stronger callus formation [22,23]. This phenomenon has also been investigated in cervical interbody fusion procedures [24]. Some authors even suspect that supraphysiologically stiff pedicle screw–rod constructs may trigger adjacent segment disease, and they argue that the optimum degree of stiffness to promote fusion is rather achieved with less stiff, dynamic stabilization of the lumbar spine [25,26]. Furthermore, Kim et al. [27] have identified CL augmentation as a driver of pseudarthrosis and have speculated that CLs could potentially displace volume for fusion mass development.

CL augmentation may, however, play a role in multilevel fusion constructs with severe AR instability, such as nonsegmentally instrumented multilevel constructs, multiple facetectomies, and unstable three-column fractures. For instance, Brodke et al. [5] observed that CL augmentation did not significantly impact AR stiffness in single- and two-level instrumentation but led to increased torsional stiffness in a three-level (L2–5) construct. Furthermore, Dick et al. [8] found that the addition of one or two CLs did not produce changes in FE, LB, and AC, but that AR was reduced by 44% with one CL and an additional 26% with a second CL. This high increase in torsional stiffness stands in contrast to our discrete findings, but may be explained by their testing model: polyurethane L3–L4 bone models with a space of 45 mm between the two vertebrae, representing a corpectomy as the worst-case scenario of instability. The conclusions drawn by our study on lumbar single-level constructs can therefore not be translated to multilevel instrumentations, for which the benefits of CL use remain unclear.

### Clinical significance

Based on our data, we believe that CL usage in a single-level lumbar fusion construct might have marginal benefits for unisegmental lumbar instrumentation constructs. The small reduction in AR-ROM must be weighed against potential disadvantages of CL implantation, such as increased surgical time, a larger surface for potential hardware-bound pathogens, and higher implant costs [14,28]. We translate the findings of our study into the following recommendations: 1) CL augmentation should not be applied in single-level lumbar instrumentations, independently of the extent of decompression and instrumentation level; and 2) CL augmentation should be reserved for rotatory-unstable long-fusion constructs, such as after a corpectomy or pedicle subtraction osteotomy. However, such scenarios were not the objectives of this study, which focused on destabilization of a single spinal segment by decompression procedures only.

### Limitations

This biomechanical cadaveric analysis entails some limitations: The isolated load applications on single-level segments in FE, LB, AR, AS, LS, and AC can only roughly be translated to the complex motion patterns and force distributions of a human spine *in vivo*. The semiconstrained setup was favored over completely unconstrained or constrained setups because the translational freedom in x-y plane allows force couples and pure compressive forces along the z-axis; in our opinion, that setup most adequately simulates *in vivo* kinematics [12,29]. However, other authors have suggested completely unconstrained test setups to allow coupled motions in all dimensions [13,30]. Further, the recommendations drawn in this manuscript are not driven by the data from this study alone, but in context with results published by previous studies [5,8,15,17,19,20].

## Conclusions

CL augmentation reduces AR-ROM by 7–12% in single-level instrumentation of the lumbar spine, with the effect increasing in tandem with the use of more extensive decompression techniques. In light of the discrete absolute changes, CL augmentation may be warranted for highly unstable situations only, rather than for standard unisegmental lumbar instrumentation constructs.

## Informed Patient Consent

The authors declare that informed patient consent was taken from all the patients.

## Declaration of Competing Interest

This study did not receive any financial support. “The authors declare that they have no known competing financial interests or personal relationships that could have appeared to influence the work reported in this paper.”
